# Unveiling Nanoparticles: Recent Approaches in Studying the Internalization Pattern of Iron Oxide Nanoparticles in Mono- and Multicellular Biological Structures

**DOI:** 10.3390/jfb15060169

**Published:** 2024-06-19

**Authors:** Teodora Eliana Petcov, Mihai Straticiuc, Decebal Iancu, Dragoș Alexandru Mirea, Roxana Trușcă, Paul Emil Mereuță, Diana Iulia Savu, George Dan Mogoșanu, Laurențiu Mogoantă, Roxana Cristina Popescu, Verena Kopatz, Sorin Ion Jinga

**Affiliations:** 1 Department of Bioengineering and Biotechnology, Faculty of Medical Engineering, National University for Science and Technology Politehnica of Bucharest, 1–7 Gheorghe Polizu Street, 011061 Bucharest, Romania; teodora.petcov@yahoo.com (T.E.P.); sorin.jinga@upb.ro (S.I.J.); 2 Department of Applied Nuclear Physics, National Institute for R&D in Physics and Nuclear Engineering “Horia Hulubei”, 30 Reactorului Street, 077125 Magurele, Romania; mihai.straticiuc@cern.ch (M.S.); decebal.iancu@nipne.ro (D.I.); dragos.mirea@nipne.ro (D.A.M.); paul.mereuta@nipne.ro (P.E.M.); 3 National Research Center for Micro and Nanomaterials, National University for Science and Technology Politehnica of Bucharest, 313 Splaiul Independentei, 060042 Bucharest, Romania; truscaroxana@yahoo.com; 4 Department of Life and Environmental Physics, National Institute for R&D in Physics and Nuclear Engineering “Horia Hulubei”, 30 Reactorului Street, 077125 Magurele, Romania; 5 Department of Pharmacognosy & Phytotherapy, Faculty of Pharmacy, University of Medicine and Pharmacy of Craiova, 2 Petru Rareș Street, 200349 Craiova, Romania; george.mogosanu@umfcv.ro; 6 Research Center for Microscopic Morphology and Immunology, University of Medicine and Pharmacy of Craiova, 2 Petru Rareș Street, 200349 Craiova, Romania; laurentiu_mogoanta@yahoo.com; 7 Department of Radiation Oncology, Medical University of Vienna, 18–20 Waehringer Guertel Street, 1090 Vienna, Austria; verena.kopatz@meduniwien.ac.at

**Keywords:** nanoparticles internalization, iron oxide nanoparticles (IONPs), internalization methods, optical microscopy, scanning electron microscopy (SEM), energy dispersive X-ray spectroscopy (EDX), particle induced X-ray emission (PIXE), monocellular models, multicellular models, biodistribution

## Abstract

Nanoparticle (NP)-based solutions for oncotherapy promise an improved efficiency of the anticancer response, as well as higher comfort for the patient. The current advancements in cancer treatment based on nanotechnology exploit the ability of these systems to pass biological barriers to target the tumor cell, as well as tumor cell organelles. In particular, iron oxide NPs are being clinically employed in oncological management due to this ability. When designing an efficient anti-cancer therapy based on NPs, it is important to know and to modulate the phenomena which take place during the interaction of the NPs with the tumor cells, as well as the normal tissues. In this regard, our review is focused on highlighting different approaches to studying the internalization patterns of iron oxide NPs in simple and complex 2D and 3D in vitro cell models, as well as in living tissues, in order to investigate the functionality of an NP-based treatment.

## 1. Introduction

The internalization of nanosized medical devices into biological structures is a critical process for evaluating their efficiency, as well as safety for usage [1,2,3]. In the case of nanostructured systems employed in therapeutic applications targeting the death of eukaryotic or prokaryotic cell populations, it is important for the systems to be internalized into the target cells [1,4] so that the device can exert its therapeutic effect and achieve maximum efficiency [5].

Iron oxide nanoparticles (IONPs) have been extensively applied in clinical oncologic management, from diagnosis (contrast substance in MRI-Resovist [6,7,8]) to treatment (magnetic hyperthermia- NanoTherm, an amino-silicone-coated- IONPs from MagForce company [8], as well as iron-deficient anemia medication (Ferinject, Venofer and Maltofer iron conjugates from ViforPharma company [8]; Manofer, Diafer, and Cosmofer, which are iron dextran colloids from Pharmacosmos company [9]; Feraheme, which is an iron polyglucose sorbitol [10], etc.)). Properties of IONPs such as biocompatibility with living tissues [11,12], the existing physiologic iron transport [12,13], and metabolic pathways [13,14,15,16], as well as magnetic properties [16,17,18], make them essential candidates in medical applications requiring the active targeting of certain cells, as well as the active targeting of their intracellular compartments. 

The internalization mechanism of the nanosystems into target cells plays a key role in the efficiency, influencing intracellular trafficking [13,15,19]. Thus, the main cellular internalization mechanisms include the diffusion mechanism, a passive and non-specific transport mechanism which does not require energy consumption [3]. Therefore, it is often undesirable because of its stochastic character, happening in both normal and tumor cells, and the main way to control this effect is through dimensional manipulation.

Energy-consuming internalization mechanisms include endocytosis, phagocytosis, and pinocytosis [3,20]. Endocytosis can occur through a recognition mechanism mediated by a receptor or membrane-level recognition, involving the formation of an invagination at the membrane site, resulting in the formation of a vesicle-like organelle in the intracellular space [3]. Two well-known endocytosis mechanisms are clathrin-mediated endocytosis and caveolin-mediated endocytosis, where proteins facilitate the formation of the vesicle from the cytoplasmic membrane and its transport into the intracellular space [3]. Phagocytosis and pinocytosis involve the formation of pseudopods that “engulf” objects of large dimensions or large volumes of extracellular fluid, including nanosized devices. Phagocytosis is a mechanism specific to immune system cells, such as macrophages, while pinocytosis can occur in all types of cells, especially in the case of tumor cells, which require a high amount of nutrients [21].

Investigations regarding the nanoparticle internalization ability into cells have been conducted using various techniques such as optical, fluorescence, and electron microscopy [22]. These techniques qualitatively highlight the locations of nanoparticles/aggregates of nanoparticles in relation to cellular organelles [23]. Electron microscopy techniques provide the ability to directly visualize internalization mechanisms by identifying characteristic morphological aspects of certain processes [24,25]. However, labeling specific proteins involved in these mechanisms with antibodies provides certainty about the type of biological process, offering a (semi-) quantitative and time-dependent estimation [26,27]. Furthermore, spectroscopy techniques allow for precise determination of the quantity of internalized nanoparticles in cells [28]. Nevertheless, these techniques do not completely replace the use of microscopy, as the visual aspect is crucial for highlighting the positions of nanosystems in relation to cellular organelles.

The interest in implementing nanotechnology in clinical applications is still high [29,30,31], especially in developing NP-based therapies for cancer management, where there is an increased demand for smart solutions able to surpass biological barriers and to target the intracellular processes of the tumor cells. Up to this point, there have been 313 clinical trials involving the implications of nanoparticles in cancer (according to Clinicaltrials.gov database, on 25 March 2024), among which 297 refer to different NP-based systems used for treatment purposes and 113 of these studies have been completed. 

Our group has been conducting comprehensive research in this field, with contributions in the development of iron oxide-based drug delivery systems (IONP_CO/DOX_) used in the radiosensitization of tumor cells [22,32,33,34], and we have studied the intracellular delivery and retention of these NP with different approaches. This article aims to initiate a discussion on the use of various microscopy and spectroscopy methods to highlight the internalization of IONP_CO/DOX_ nanosystems in unicellular, multicellular, 2D, and 3D biological structures. This review is based on our own results. 

## 2. Internalization Mechanism of Iron Oxide Nanoparticles

In oncologic management, one of the challenges of classical chemotherapy is delivering drug molecules safely and specifically to selected cell types at a therapeutically effective concentration. One potential solution to this issue is the utilization of nano-sized medical devices based on IONPs. The primary objectives of nanomedicine development are to create enhanced formulations that can target and deliver drugs in a controlled manner while minimizing toxicity and overcoming biological barriers to reach the desired target. In order to accomplish these targets, nanosystems can undergo various internalization processes [23].

The mechanism of internalization of the nanostructured system into the target cells is critical to its efficacy, as it influences how intracellular trafficking takes place [5]. There are two main mechanisms of cellular internalization: diffusion, which does not require energy consumption, and mechanisms that involve energy consumption. Given the preferential aggregation of IONPs due to their magnetic properties [35,36], they rarely undergo a passive transport mechanism such as diffusion. Moreover, because bare IONPs undergo opsonization in vivo, most of the nanomedicines based on IONP have different coatings [37], increasing the hydrodynamic diameter of the system [38,39,40].

During the interactions between IONPs and cells, it is observed that the initial stages involve the adhesion of nanoparticles to the cell membrane and their potential interaction with cell receptors. Moreover, most of the cells have receptors for Fe^2+^ ion transport, such as the Divalent Metal Transporter 1 or CD71 and Ferritin involved in transferrin import [40]. To regulate internalization events, IONP can be modified with targeting fragments, such as peptides [41,42], proteins [43,44], or antibodies [45,46], to specifically recognize receptors on the cell surface and achieve active targeting. Nanomaterials may interact with a single type of receptor, or with several simultaneously, to induce uptake. High-affinity interactions may contribute to entry or internalization, while internalization can also occur without recognition by specific receptors, being triggered by the nanosized object. Notably, cellular receptor recognition may only be involved in initial adhesion to the cell membrane without necessarily contributing to internalization. Various interactions between nanoparticles and cells are possible [47,48].

**Figure 1:**
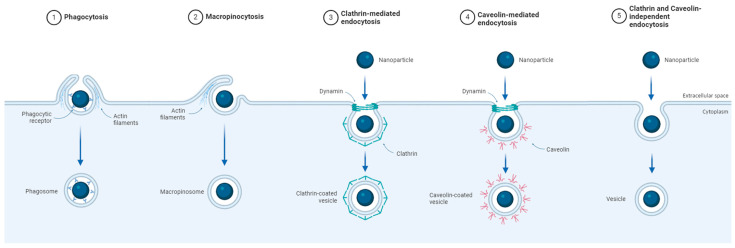
Schematic representation showing the mechanisms of nanoparticle cellular internalization such as (**1**) phazxgocytosis; (**2**) macropinocytosis; (**3**) clathrin-mediated; (**4**) caveolin-mediated; and (**5**) clathrin-and caveolin-independent pathways; scheme created with Biorender.com (accessed on 15 March 2024) [49].

This section outlines the primary energy-consuming mechanisms of cellular internalization, which include endocytosis, phagocytosis, and pinocytosis (Figure 1).

Endocytosis is a cellular process that involves the internalization of external substances or particles. This process can be facilitated by receptor recognition or driven by the properties of the cell membrane, leading to the formation of an invagination in the membrane and the subsequent generation of an intracellular vesicle. Cells have developed multiple endocytosis mechanisms to choose and organize various cargoes towards their intracellular destinations [21]. Although these mechanisms have similarities, they also have significant differences. Two well-known mechanisms of endocytosis are clathrin-mediated and caveolin-mediated endocytosis.

In clathrin-mediated endocytosis, concavities form on the cell membrane, allowing external substances to be internalized within a clathrin-coated transport vesicle. The mechanism described is considered essential for the uptake and internalization of specific molecules, including membrane proteins and growth factors [50,51]. Clathrin-mediated endocytosis can be triggered by receptor recognition, such as transferrin, low-density lipoprotein receptors, insulin receptor, G-protein receptors, etc. Targeting these specific receptors [52,53,54] can be a measure to set off the clathrin-mediated endocytosis process. Also, transferrin receptors are over-expressed in cancer cells [5], being a key targeting element of NP-based therapies. Additionally, considering the most common endocytosis mechanism, it is expected that many types of IONPs undergo this process of intracellular transport. The use of specific inhibitors has proven that fluorescent BODIPY^®^-labeled IONP can be transported through clathrin-mediated endocytosis in microglia cells [55]. Mazzolini et al. [56] proved that the transferrin in the serum protein corona covering nanoparticles might be recognized by the specific receptors generating their clathrin-mediated endocytosis. 

Additionally, caveolin-mediated endocytosis involves the formation of small depressions in the cell membrane called caveolae, which facilitate the internalization of substances into a transport vesicle known as a caveosome. This mechanism is typically associated with the uptake of lipids and specific molecules, such as steroid hormones and some bacterial toxins [51]. Caveolin-dependent endocytosis is triggered in certain areas of the plasma membrane and the lipid raft regions and can be activated by molecules such as insulin or albumin [5]. PEG-coated IONPs have been shown to be internalized preferentially through caveolin-dependent endocytosis [57,58], as well as liposome-based and extracellular vesicle-based nanoparticles [59].

On the other hand, phagocytosis and pinocytosis are processes by which a cell can internalize large particles or large quantities of extracellular fluid which contain nano-sized devices. Phagocytosis is a process specific to immune system cells, such as macrophages, which destroy foreign particles or dead cells by engulfing them. During phagocytosis, the plasma membrane emits pseudopods to engulf the particles, forming large vesicles called phagosomes. In contrast, pinocytosis can occur in almost all cell types, including tumor cells, which have an increased tendency to satisfy their nutritional needs by internalizing significant amounts of substances from the extracellular environment [19,21]. IONPs are preferentially internalized through macropinocytosis in HeLa adenocarcinoma cells [22], RAW264.7 macrophage cells [60], A-549 lung cancer cells [61], etc., due to their aggregation tendency.

Efficient and controlled entry of NPs into cells is a crucial factor for achieving high prognostic and therapeutic efficacy. The intracellular destination of NPs is vital for their successful use as vehicles for delivering specific molecules to the nucleus or other intracellular sites. However, it is important to acknowledge that achieving this remains a major challenge, as well as to agree that it is impossible to trigger a unique internalization mechanism by certain engineered nanoparticles. It is necessary to carefully tune the physicochemical properties of the NPs to optimize targeting, uptake, and cellular trafficking, as well as interactions with the cell membrane [50].

The design of IONPs can be customized to target specific cell types or specific pathways of internalization. However, one must acknowledge that these processes do not occur at 100% capability, but one can attain a higher percentage in preferential cells/processes. The characteristics of nanomaterials, such as size, charge, shape, hydrophobicity, stiffness, roughness, and surface functionalization, can be modified to influence their internalization pathways. This may result in their targeting to a specific intracellular location [4,50]. Table 1 presents recent studies that demonstrate the diverse properties of nanoparticles that may affect their cellular uptake and the methods used to prove the internalization of the NPs, with an accent on iron oxide.

## 3. Investigations of the Iron Oxide Nanoparticle Internalization Process in Conventional 2D Cell Cultures

NPs can be characterized using various techniques to determine their size, size distribution, shape, surface area, porosity, aggregation, charge, and crystallinity [76,77]. In the meantime, techniques commonly used to characterize NPs interacting with cells include Fourier transform infrared spectroscopy (FTIR) [78], energy-dispersive X-ray spectroscopy (EDX) [79], scanning electron microscopy (SEM) [80], transmission electron microscopy (TEM) [81], fluorescence optical microscopy [82], and spectroscopic measurements [83]. 

These analysis techniques have significantly contributed to the current understanding of the navigation of NPs in different cells. These methods provide a significant amount of information on the properties of NPs [84], their spatial distribution in specific cell types [85], the corresponding molecular interactions [86], and the phenotypic or genotypic effects of these perturbations [87], as well as their time dependence [88]. Recent advancements in different characterization techniques have further improved our understanding of the complexity of nanoparticle trafficking and strengthened our ability to control these cellular interactions [89].

### 3.1. Optical and Fluorescence Microscopy

The biological evaluation of nanosized medical devices, such as IONPs, is always initiated with in vitro preclinical tests. It is important to study the effect of the nanodevice on target cells, as well as on other cells in major organs where they could be transported. In the case of nanostructured systems used in antitumoral treatment, it is important to conduct a comparative study using both tumor cells (from at least two donor sources/two types of commercial tumor cells) and normal cells that may be in the vicinity of the tumor tissue/host tissue.

Visualization of cells using different microscopy techniques is important for both morphological and structural characterization of the samples. Resolution is the most crucial parameter, describing the ability of the equipment to distinguish details in the sample, and is defined as the minimum distance between two distinct points in the sample that can be observed as separate entities by the observer [90]. This property is determined by the optical system characteristics, such as the numerical aperture of the objective used, but it is also influenced by the wavelength of the light source used [90].

Optical microscopy is one of the most accessible techniques for visualizing cell cultures or tissue sections. The visualization of these structures is possible due to the small thickness of the specimens (tens of µm to sub-10 µm), with an ideal lateral resolution for an optical microscope being a minimum of 200 nm, with an axial resolution of 500 nm [91]. Biological samples appear transparent, and their delineation, or certain cellular organelles such as the nucleus, can be observed due to their refractive index being different from that of the cultivation substrate [92]. To highlight specific cellular organelles (within the resolution limits imposed by the technique/equipment), specific stains or an antigen-antibody-based labeling system are used, where the antigen is represented by specific molecules from cellular substructures. This procedure, applied to isolated cells, is called immunocytochemistry and involves marking certain proteins of interest with a primary antibody against the respective protein (e.g., anti-tubulin antibody that specifically reacts with tubulin in the cellular cytoskeleton). Subsequently, a secondary antibody is used against the primary antibody, conjugated with an enzymatic label that catalyzes a color reaction in the substrate (e.g., the primary antibody is derived from a mouse, so the secondary antibody must come from another animal, and it must also specifically react with primary mouse antibodies).

**Figure 2:**
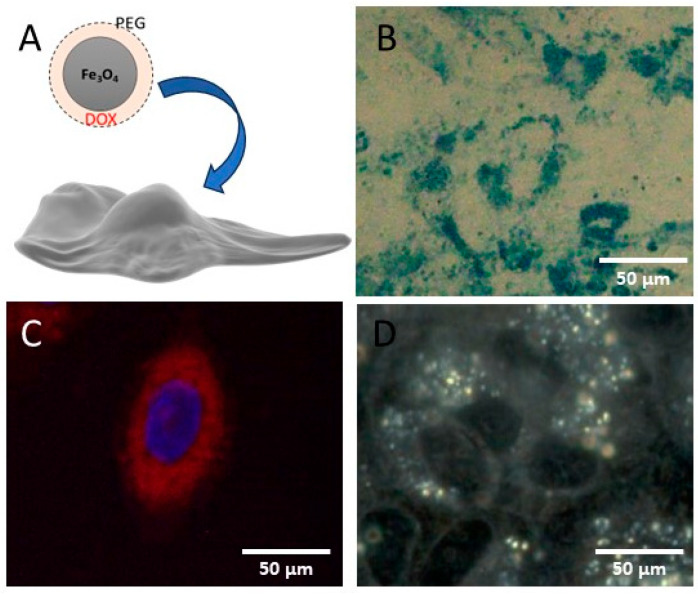
Employment of optical microscopy techniques to prove IONP_DOX_ nanoparticle internalization in SW1353 chondrosarcoma cells: (**A**) chondrosarcoma cells were incubated for 16 h with the core–shell doxorubicin-loaded nanoparticles; (**B**) optical microscopy image of treated cells where a Prussian Blue reaction between iron in the NPs and ferrocyanide led to a blue coloration of iron oxide nanoparticle aggregates in the cells; (**C**) fluorescence image revealing a red signal from doxorubicin in the nanoparticles and blue signal from Hoechst-stained nuclei; (**D**) dark field image revealing the light reflection due to iron oxide nanoparticles; image adapted from Ref. [34].

Visualization of nanoparticles in optical microscopy is not possible if they do not have adequate concentrations and do not aggregate in the intracellular environment, so these clusters exceed the resolution limit. Often, they are visualized due to light reflection, but specific staining methods can also be used, such as Prussian Blue for IONPs [22] (Figure 2) or the reaction with NaCl for gold-citrate nanoparticles [93].

The immunocytochemistry technique can be enhanced using fluorescence microscopy. This is achieved through a procedure similar to the one described earlier, but in this case, secondary antibodies are conjugated with fluorophores. By using a confocal system with monochromatic radiation, lateral resolution can reach up to 180 nm [94]. Moreover, direct labeling of nanoparticles with fluorophores can increase their detection sensitivity and specificity, making it easier to monitor their distribution in cellular substructures. It also allows for the quantification of the associated fluorescence signal intensity for (semi-) quantitative determinations [95,96,97,98]. Furthermore, multiplexing is possible, enabling the simultaneous visualization of multiple fluorophores with different excitation–emission properties [28,99,100,101], as well as multiplex engineered nanoparticles, such as iron oxide core@ multi-shell NPs, which are able to emit fluorescence at different wavelengths [102]. The technique also provides the opportunity for real-time monitoring using specific fluorophores for labeling living cells/organelles in living cells (known as cell trackers) [103,104]. 

According to Figure 2, the optical microscopy (Figure 2B) and fluorescence microscopy (Figure 2C) images depict the human chondrosarcoma SW1353 tumor cells after being exposed to treatment with IONPs (500 µg/mL IONP_DOX_ incubated for 16 h). IONPs could be colored due to a specific reaction between potassium hexacyanoferrate trihydrate and Fe under acidic conditions, resulting in Prussian Blue stain [33] (Figure 2B).

An example of fluorescently labeled nanoparticles interacting with human chondrosarcoma SW1353 cells [34] is illustrated in Figure 2C. Here, the specific Hoechst fluorophore was used to highlight the cell nucleus, making it visible in fluorescence microscopy at excitation/emission wavelengths of 352 nm/454 nm [105]. The chemotherapeutic drug doxorubicin (DOX) was used to assess the potential of an IONPs-based system in the controlled release and radiosensitization of human chondrosarcoma tumor cells. The observation of DOX was possible due to its inherent fluorescence properties [106].

Additionally, dark-field microscopy is a special form of optical microscopy that has significant potential for fast, efficient, accessible, and non-invasive imaging of a wide range of nanosized materials. It can be considered as a viable alternative to more complex and expensive microscopy techniques, such as electron microscopy, for the rapid identification of nanoparticles in biological structures (cells, tissues) [107]. This technique has been extensively used for spectral characterization of nanoscopic particles, as it detects light scattered by a sample without the need for fluorescent labeling to detect a single molecule. However, further exploration is required in order to fully understand its sensitivity to variations in molecular density [108].

To demonstrate the behavior of SW1353 chondrosarcoma cells after Fe_3_O_4_@PEG 6K/DOX treatment, dark field microscopy combined with a hyperspectral imaging module was used [34]. This imaging combination allows for analysis at the single-cell level, providing details on the nucleus and nanoparticle internalization into the cytoplasm. Hyperspectral microscopy images were recorded of SW1353 cells treated with IONP_DOX_ for 16 h (Figure 2D). The images reveal the presence of nanoparticles that were internalized into the cytoplasm of the tumor cells and located in the peri-nuclear area, shown as bright aggregates [34].

### 3.2. Electron Microscopy

Utilizing electron microscopy techniques to highlight the internalization phenomenon of nanosystems in biological structures has the major advantage of the high resolution offered by this type of technique, facilitating the ability to visualize nanoparticles at a subcellular and nanoscale level [24,28]. Thus, electron microscopy techniques can reveal lower concentrations of nanoparticles, as well as non-aggregated nanoparticles. Moreover, electron microscopy can highlight cellular morphological details that provide information about the internalization process of nanosystems in biological structures [109,110]. The electron microscopy equipment usually includes attached spectroscopy systems such as EDX or selected-area electron diffraction, providing the possibility of dual analysis through elemental mapping of morphological elements in the biological specimen [28].

**Figure 3:**
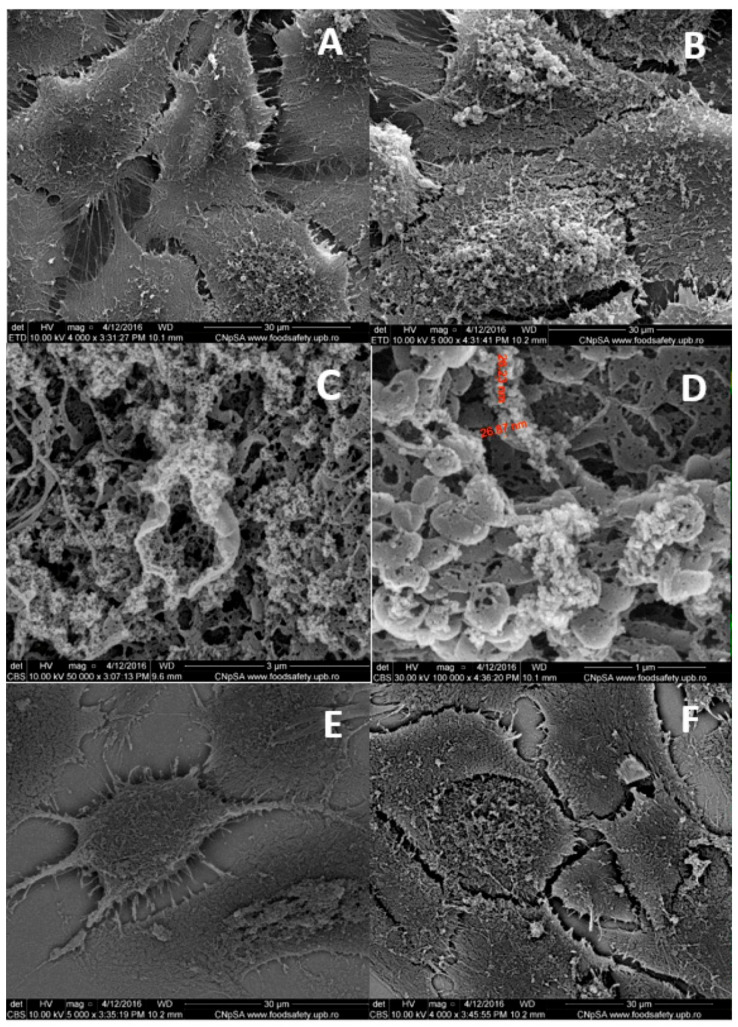
Scanning electron microscopy (SEM) images for human MG63 osteosarcoma cells incubated for 48 h: (**A**,**E**) negative control and (**B**–**D**,**F**) with 500 ppm doxorubicin-conjugated iron oxide nanoparticles; images were acquired from secondary electrons (**A**–**D**) and back-scattered electron (**E**,**F**) processing; image adapted from Ref. [28].

SEM provides an overview of the cellular internalization process of nanosystems, benefiting at the same time from the increased resolution offered by this technique. The analysis mode of backscattered electrons contributes to a surface compositional characterization (Figure 3D–F). Additionally, by simultaneously performing spectral mapping, information can be obtained from the depth of the biological specimen, depending on the parameters of electron acceleration provided by the SEM equipment [111].

Figure 3 highlights backscattered electron microscopy images in comparison to classic secondary electron microscopy images. Images acquired in the secondary electron analysis mode emphasize morphological aspects characteristic of the internalization process through micropinocytosis (Figure 3B–D), while images acquired in the back-scattered electron analysis mode reveal the compositional identification of nanosystems (Figure 3E,F). Images were recorded following 48 h of incubation of cancer cells (MG63 human osteosarcoma) with 500 ppm doxorubicin-conjugated IONPs [28]. SEM images acquired in back-scattered mode showcase elements with higher atomic number in white tones, while elements with lower atomic numbers are rendered in dark tones. Thus, all carbon-based organic matter in the cell will be revealed in dark tones in a backscattering mode SEM, while IONPs should appear like white aggregates. Scanning electron microscopy can exhibit details of the plasma membrane (structure and morphology) that can reveal important information regarding the interaction of the nanoparticles and the cell membranes, delineating the internalization mechanism. In the case of IONPs, our group has reported SEM images showing the ruffling of the plasma membrane, which is characteristic of macropinocytosis, as well as the specific macropinosome initiation [28].

### 3.3. Quantitative Spectroscopic Determinations

Quantitative determination of internalized nanoparticles in cellular structures can be made possible through various methods that rely on measuring the radiation signal of marked NPs (with fluorophores [95,96,97,98] or radioactive isotopes [112,113,114]). However, the methods with the highest accuracy remain those involving spectrometry measurements. One of the most popular techniques for assessing the quantity of internalized NPs in biological structures is inductively coupled plasma mass spectrometry (ICP-MS) [115,116], which offers a detection limit up to the order of parts per trillion (ppt) [117] and involves digesting biological structures exposed to NP treatment, followed by evaluating the elemental composition of the samples [118,119]. Single-cell ICP-MS is a rapid method for the detection of metal contents in a cell suspension, with extensive applications in toxicology studies [120]; however, the resolution (mg/L level) [119] is quite inappropriate to give a precise estimation of NPs internalization efficiency, which is an important parameter in the estimation of the nanotherapeutic efficiency [121].

Our group has applied a method based on particle-induced X-ray emission (PIXE) to measure the intracellular content of IONPs in tumor cell suspensions deposited on thin Mylar targets [28]. PIXE is a nuclear spectrometry technique used in the elemental analysis of samples [122]. Still, the method is not widely used in cellular internalization studies of nanoparticles due to its high cost, requiring the use of a particle accelerator [76,80]. PIXE is widely considered to be a non-destructive analysis technique [28,123,124], and its detection limits can reach the order of parts per million (ppm) [125,126]. The technique involves “bombarding” the sample under analysis (cells incubated with NPs) with charged particles (protons or ions), resulting in the emission of characteristic X-rays of elements in the sample. Then, these characteristic X-rays are detected and used to determine the presence of certain elements and their concentrations. Quantitatively, these determinations can be achieved by comparing them to an internal standard (cellular suspension with a known concentration of NPs added).

**Figure 4:**
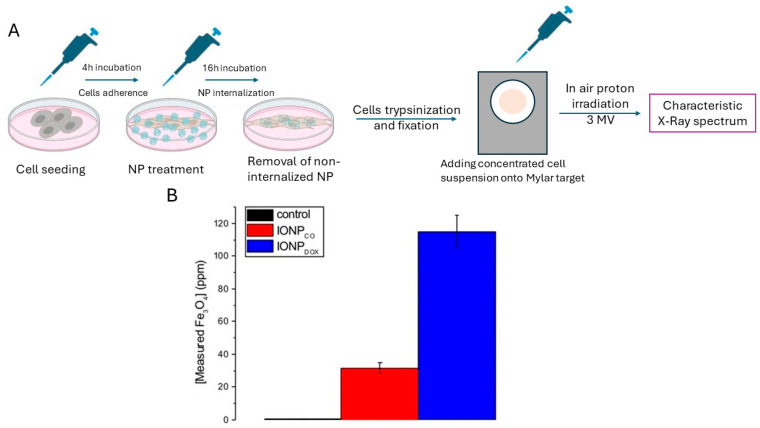
Particle-induced X-ray emission (PIXE) for human cervical adenocarcinoma HeLa cells incubated for 16 h with IONP_CO_ and IONP_DOX_ nanoparticles; (**A**) protocol for PIXE sample preparation; scheme created with Biorender.com (accessed on 15 March 2024). (**B**) Quantity of internalized iron oxide nanoparticles; image adapted from Ref. [34].

The example presented in Figure 4 assumes the determination of the internalization of IONPs in tumor human adenocarcinoma HeLa cells. Cells were seeded and incubated for 4 h to allow for attachment, followed by incubation with 100 ppm IONP_CO_ and IONP_DOX_. Incubation with NPs lasted for 16 h to prevent dilution of intracellular NP content through cell division. This time interval is shorter than the doubling time of all cell lines used. After incubation with NPs, the supernatant (non-internalized NPs) was removed by washing. The obtained sample was fixed and subjected to PIXE analysis (for treatment scheme, see Figure 4A). The internalization efficiency could be calculated by dividing the measured quantity of NPs by the administered quantity of NPs and utilizing percentage-wise calculation. This result is utterly important when designing an anti-cancer treatment based on IONPs and gives us an indication of the safe dosage of NPs (in normal cells) while still having a high internalization efficiency in the targeted tumor cells. 

**Figure 5:**
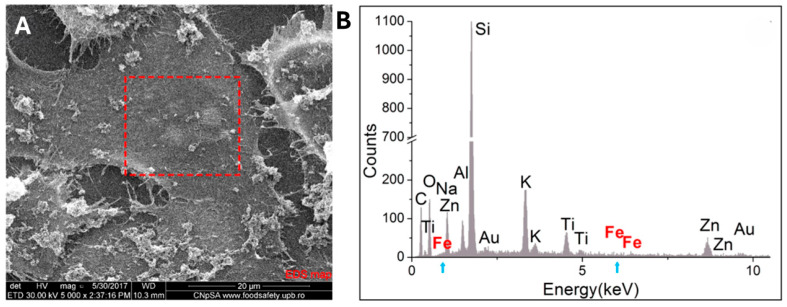
Energy-dispersive X-ray spectroscopy (EDX) for human MG63 osteosarcoma cells incubated for 24 h with gemcitabine-conjugated iron oxide nanoparticles: (**A**) scanning electron microscopy image of the area subjected to EDX analysis; (**B**) EDX spectrum of the analyzed area delimited by a red rectangle in (**A**); the peaks corresponding to the energy levels of Fe characteristic X-rays are highlighted in red and with blue arrows; image adapted from Ref. [111].

**Figure 6:**
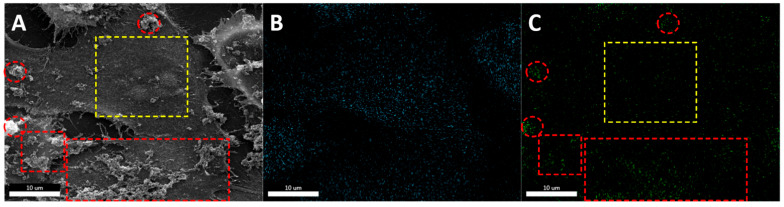
Mapping energy-dispersive X-ray spectroscopy (EDX) for human MG63 osteosarcoma cells incubated for 24 h with gemcitabine-conjugated iron oxide nanoparticles: (**A**) scanning electron microscopy image acquired using secondary electrons signal (red circles and squares—extracellular NPs, yellow square—area with no extracellular NPs); (**B**) carbon elemental mapping of (**A**); (**C**) Fe elemental mapping of (**A**); image adapted from Ref. [111].

EDX was undertaken to determine the presence of iron (Fe) on a specific area of a cell where IONPs had been confirmed through SEM images. The obtained values were restricted to the scanned area (red square in Figure 5), offering quantitative elemental determination at the level of a single cell, but not in the entirety of the cell volume [127]. By integrating analytical methodologies with cartography, one can explore the spatial arrangement of constituents within cellular entities, as well as their specific allocation within diverse cellular organelles or compartments [28]. The distribution of elements in a SEM image is emphasized in Figure 6 in osteosarcoma cells (MG63) incubated with Gemcitabine-conjugated IONPs [28,111]. Following mapping, the elements carbon (B) and iron (C) were identified. The mapping of iron revealed areas with increased signal (Figure 6C), overlapping the IONP clusters in the high-resolution SEM image (Figure 6A, red circles and squares). Moreover, the information obtained from secondary electrons SEM (topographical details) and EDX mapping (compositional differences in the sample up to 1–10 µm depth, elemental composition and concentration in the sample: 2D representation) can reveal information on the nanoparticles’ internalization mechanism, their interaction at the plasma membrane level, as well as their location in the extracellular or intracellular compartment [28,111]. This approach was reported by our group for tumor cells exposed to drug delivery systems based on IONPs [28,111]. 

## 4. Immunohistological Studies for Complex Biological Structures

In vitro 3D cultures are systems in which cells are grown under simulated conditions outside the human body within a three-dimensional structure. This cellular arrangement provides biomimetic conditions, allowing cells to interact with each other and their surrounding environment (the extracellular matrix, ECM), resulting in cells exhibiting phenotypes closely resembling real-life conditions. This setup enables the investigation of cellular behavior with high accuracy, capturing cells’ physiological responses.

In nanotechnology, these 3D cultures have been employed in applications such as: (1) toxicity studies [128,129,130,131], (2) understanding cellular mechanisms in response to interaction with nanostructured (medical) devices [132,133], (3) tissue engineering [134,135,136], (4) controlled NP-based delivery systems [137,138,139], and (5) modeling microenvironments like the tumor microenvironment for investigating innovative treatment solutions [140,141,142].

The technology for obtaining in vitro 3D cell cultures has expanded in recent years and encompasses the following main classes of methods [143]: (1) suspension cultures utilizing non-adherent plates (liquid overlay method or drop casting) or rotating culture systems (reactors) [144], (2) methods using 3D scaffolds (degradable and non-degradable) [145], (3) (bio)printing 3D methods [146], and (4) microfluidic systems [147].

**Figure 7:**
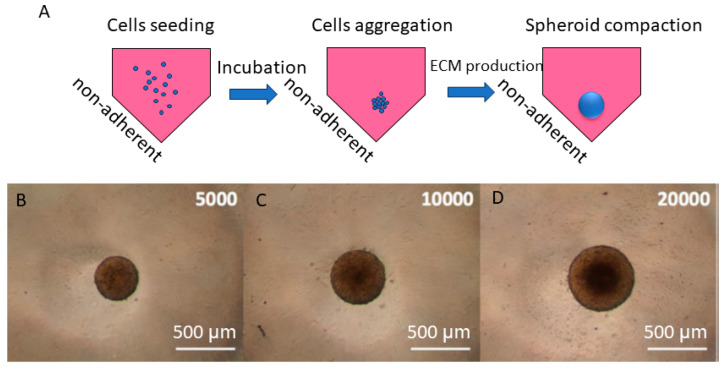
Generation of in vitro 3D squamous cell carcinoma cell models: (**A**) the principle of the liquid overlay method; (**B**–**D**) morphology of human squamous cell carcinoma FaDu spheroids obtained through the liquid overlay method with (**B**) 5000 cells/spheroid, (**C**) 10,000 cells/spheroid, and (**D**) 20,000 cells/spheroid at 3 days post-seeding; image adapted from Ref. [33].

In Figure 7, the method for obtaining spheroids through the liquid overlay technique is illustrated. This method is very common for obtaining 3D tumor cell models [148,149,150] and involves the use of culture plates with anti-adherent coating and a conical or round shape of the wells (Figure 7A). Cells are seeded in such plates at appropriate densities, followed by incubation under standard temperature and humidity conditions for several days (up to 3 days) to allow for cell aggregation and spheroid compaction through the secretion of ECM (Figure 7A). Figure 7B–D depict the morphology of human squamous carcinoma FaDu spheroids obtained through the liquid overlay method and the dimensional evolution for different initial cell densities: 5000, 10,000, and 20,000 cells/spheroid. Such methods are reliable, reproducible, and easy to apply for obtaining large quantities of spheroids used in monitoring the interactions of NPs with cellular models. They are applicable for quantitative assessments of cell viability following interactions with NPs, methods that require a large number of spheroids to promote statistically reliable results.

The key advantages of 3D in vitro cultures compared to classical 2D models in adherent culture vessels is that they provide more physiologically relevant responses for nanoparticle–cell interaction assessments, especially regarding tissue penetration and internalization [151,152,153,154,155]. Cells benefit from a 3D architecture due to the organization of the ECM in a manner closer to physiological conditions [156,157]. Moreover, this spatial organization facilitates the secretion of ECM protein components by cells and stimulates the formation of a complex network of carbohydrates and proteins, providing structural support to cells [157]. Compositionally, the ECM secreted by cells in 3D cultures is similar to that found in living tissues. Thus, cells cultured in such 3D systems are also stimulated to differentiate. Intercellular interactions are facilitated by a 3D microenvironment that allows communication from all directions [156]. This organization promotes multicellular heterogeneity, not only in monocellular but also in multicellular cultures. By cultivating cells in a 3D architecture, a physiological gradient of oxygen, nutrients, and signaling molecules is facilitated, mimicking the gradients present in living tissues. These gradients stimulate cell migration, differentiation, and the cellular response to various stimuli, such as NP treatment.

**Figure 8:**
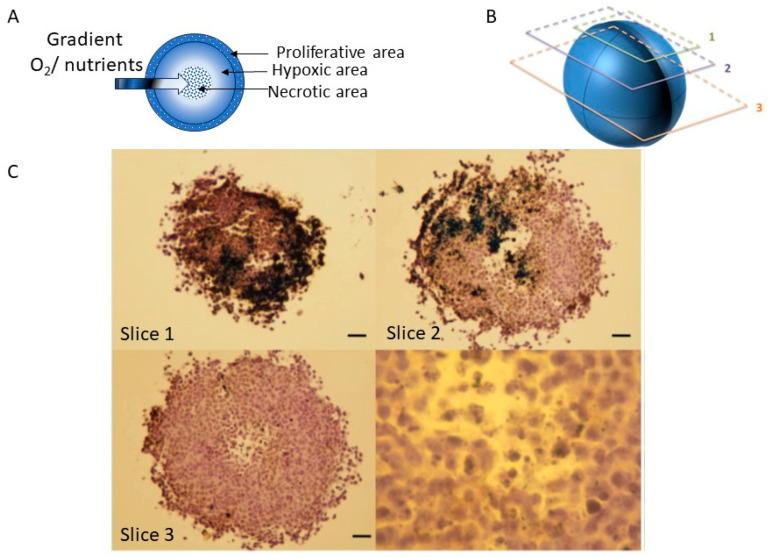
Morphological characterization of human squamous cell carcinoma FaDu spheroids obtained through the liquid overlay method at 3 days post-seeding: (**A**) schematic representation of the main morphological and functional zones of the spheroids; (**B**) schematic representation of the cross-sectional slicing approach through spheroids for morphological and functional characterization; (**C**) optical microscopy images for cross-sectional slices through FaDu human squamous cell carcinoma spheroids, hematoxylin violet, and brown nanoparticles; slices 1–3 correspond to the highlighted zones in (**B**); image adapted from Ref. [33].

In particular, the response of tumor cells in 3D cultures is closer to real behavior, stimulating the formation of a biomimetic tumor microenvironment (Figure 8). The spontaneously formed oxygen and nutrient gradient in spheroid-like cellular models simulates the concentrations of these vital elements in the in vivo environment (Figure 8A). Moreover, due to this phenomenon, cells exhibit different phenotypes characteristic of predefined zones (Figure 8) [33]: (1) the proliferative zone, located at the spheroid’s periphery, where cells have access to nutrients and oxygen from the environment and cells exhibit an accelerated metabolism and a characteristic cell division rate for the simulated tumor model; (2) the hypoxic zone, located after the proliferative zone, where cells have a slowed metabolism and are in a cell cycle arrest phase, with reduced access to nutrients and oxygen; and (3) the central necrotic zone, where cellular debris accumulates resulting from necrosis caused by the depletion of vital elements.

A depiction of these zones can be achieved using classical immunohistochemical methods for characterizing tumor tissues collected from living organisms. After obtaining 3D tumor cell cultures through methods such as liquid overlay, they are harvested, fixed, and dehydrated using techniques similar to those utilized in the routine processing of tissue specimens from different organs [33]. The 3D cultures are then embedded in solid blocks (paraffinized), and thin sections with thicknesses up to 3–4 μm are made to allow for the analysis of cellular structures (Figure 8B,C). After an extensive deparaffinization and rehydration process, cells can be stained using various standard histology stains (e.g., haematoxylin-eosin), and specific cells/structures can be highlighted through specific antibody labeling [33].

In Figure 8C, cross-sectional views of human squamous cell carcinoma FaDu spheroids obtained through the liquid overlay method are shown. To characterize these spheroids morphologically and compositionally, sections obtained at different depths (Figure 8B) were specifically labeled [33].

Once the tumor spheroids have been treated with NPs, they interact with the tumor cells at the top of the 3D culture and, due to their weight and diffusion into the culture medium in the well, the NPs gradually move downwards via sedimentation. Therefore, proliferative cells at the spheroid’s upper extremity are in direct contact with NPs, and their transport towards the hypoxic region is strictly determined by the hydrodynamic and surface properties of the designed NPs, as well as the incubation time. The access of NPs to hypoxic zones is crucial for the success of the intended treatment. 

Static methods for obtaining and maintaining in vitro 3D cell cultures such as spheroids (e.g., the liquid overlay method) have the disadvantage of offering transport based solely on diffusion. In order to mitigate the transport effect in 3D cell cultures, micro-channel systems [158,159,160,161] or stirring bioreactors can be used [161,162,163,164]. Stirred bioreactors promote uniform circulation of the culture medium around spheroids [164], while microchannel systems facilitate the controlled and precise transport of nutrients and oxygen to cells [165]. These two systems offer a promising approach to enhancing the growth and operating conditions of 3D cell cultures.

**Figure 9:**
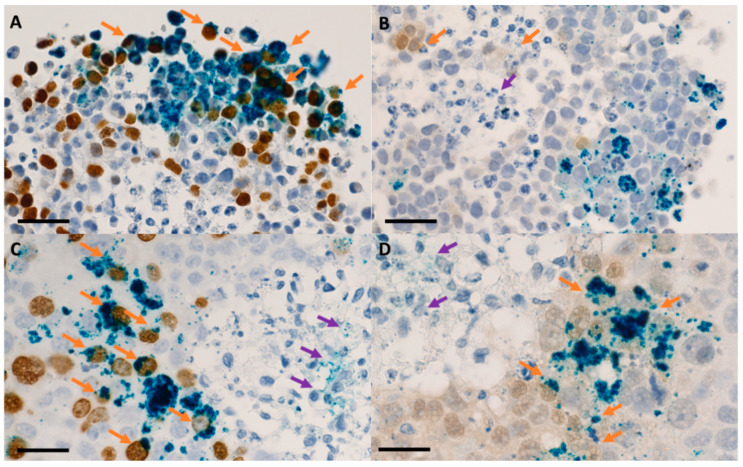
Optical microscopy images of sections in tumor spheroids that were previously incubated with 200 μg/mL Fe_3_O_4_@PEG 6K/DOX nanoparticles for 48 h. Details: (**A**,**B**) HeLa human cervical adenocarcinoma cells; (**C**,**D**) FaDu human squamous carcinoma cells and immunohistochemical staining of proliferative areas using bromodeoxyuridine (BrdU); (**A**,**C**) and hypoxic areas using pimonidazole (Pimo) (**B**,**D**). Light blue—hematoxylin, dark blue—iron oxide, BrdU—dark brown, Pimo—light brown; scale bar—25 μm. Orange arrows indicate aggregates of iron oxide nanoparticles internalized in proliferative/hypoxic cells, and purple arrows indicate nanoparticles present in the necrotic core Ref. [165].

Performing longitudinal sections in the direction of the concentration gradient can provide additional information regarding the penetration ability of the NPs into the tumor spheroid. Additionally, immunohistochemical labeling of specific cells can indicate further aspects of the types of cells interacting with the NPs. By pre-incubating spheroid cultures with bromodeoxyuridine (BrdU) and then with an anti-BrdU antibody, proliferating melanoma cells in the S phase of the cell cycle can be highlighted (Figure 9A,C—cells in dark brown). Accordingly, the hypoxic zone can be highlighted by spheroid treatment with pimonidazole (Pimo) and an anti-Pimo antibody (Figure 9B,D, cells in light brown). Necrotic centers were only observed in the cases of spheroids obtained from human squamous cell carcinoma FaDu through the liquid overlay method, as this structure only appears in larger or more dense spheroids. 

In Figure 9, cross-sectional views through HeLa human cervical adenocarcinoma spheroids (Figure 9A,B) and FaDu human squamous carcinoma spheroids (Figure 9C,D) incubated for 24 h with PEG-conjugated IONPs are shown. These sections were processed to highlight proliferative cells (Figure 9A,C, dark brown) and hypoxic cells (Figure 9B,D, light brown). However, creating longitudinal sections, as well as those transverse to the concentration gradient direction, is more of a stochastic process that cannot be precisely controlled following the histological preparation procedure of spheroids.

Conducting cellular internalization studies in 3D cellular models can be more challenging, because cells in the 3D microenvironment have a more compact morphology compared to the spread-out configuration in classical 2D models. Using classical immunohistochemical techniques, internalization of NPs at the submicrometric and subcellular levels can be appreciated by their relative positioning to the cell nucleus or cytoplasm (Figure 9) [33]. The resolution limit of optical microscopy can be overcome by using fluorescence/confocal microscopy techniques. However, for these techniques, the nanoparticles used must have fluorescent properties or be functionalized/incorporated with various fluorophores. In this way, the NPs could be highlighted at a higher resolution. Furthermore, due to the ability to scan along the Z-axis, the 3D cell model would not require prior preparation through sectioning, but could be scanned entirely (depending on its dimension). In terms of providing detailed information about possible interactions at the level of cellular organelles with nanoparticles, electron microscopy techniques remain the most precise and can be applicable in the case of cellular spheroids [166,167].

Although the complexity of in vitro 3D cell models has increased significantly with advancements in tissue engineering research, complete anatomically and functionally representative models in an artificial environment outside the living organism have not yet been developed. Moreover, the coexistence and interaction of complex systems within the human body can particularly influence the outcome of experiments evaluating pharmaceutical systems, such as IONPs [168,169]. This is mainly due to the nature of the proposed treatment, such as introduction into systemic circulation to passively or actively target the tumor tissue. Thus, the nanosystem can simultaneously interact with multiple biological structures in the body. Therefore, following favorable in vitro characterization of such nanosystems, it remains imperative to conduct in vivo tests on representative animal models for preclinical validation of the proposed treatment.

To achieve effective cytotoxic treatment, it is crucial to ensure the proper concentration and distribution of NPs within cancer tissue when creating a nanostructured medical device [170]. It is also essential for these NPs to exhibit low toxicity in both short- and long-term evaluation [11,171,172]. These particles have the potential to reach various parts of the body, including the connective tissue, liver, spleen, lymph nodes, lungs, and central nervous system [100]. The presence of NPs, as well as any induced histopathological alteration, can be evidenced through a thorough immunohistochemical preparation of collected tissue samples.

The significant impact of the reticuloendothelial system on the overall distribution of intravenously administered IONPs is worth noting. The immune system has various mechanisms to eliminate foreign particles. When colloidal particles enter the circulation, they are directed to specific organs. Depending on the NPs’ hydrodynamic properties and surface chemistry [173,174], they might interact with opsonins, which are self-proteins that facilitate the recognition of foreign particles by the immune system and cover the particles, followed by specific elimination by macrophages. This process, known as opsonisation, enhances the interaction of antigens with immune cells and can lead to the NPs’ subsequent elimination. NPs are often perceived as foreign particles, which leads to the binding of opsonins to them, thereby facilitating phagocytosis. 

Upon entering the bloodstream, highly mobile proteins initially attach to the NPs, forming soft coronas. As time passes, higher affinity proteins replace some of these proteins, creating hard coronas. The hard coronas then bind to receptors on the surfaces of phagocytic cells, initiating internalization and digestion of the particles [175,176,177]. Engineered IONPs have been designed in order to modulate the composition of protein coronas [173], and also to increase the systemic circulation time of the nanosystem [178].

Recent studies have demonstrated that NPs are eliminated from most organs after 100 days following injection, without any lasting toxicity [179,180]. It has been observed that the size and surface coating characteristics of the NPs ultimately determine their tissue deposition [181,182,183]. Additionally, smaller NPs (20 nm or less in diameter) have been found to penetrate tumor tissue more effectively [183,184]. It is worth noting that smaller particles have a tendency to remain in circulation for longer periods [185,186]. This is because larger NPs are more readily sequestered by Kupffer cells in the liver [171].

The properties of NPs, such as chemical reactivity, adsorption, and agglomeration, can significantly influence their half-life in a given medium [187,188]. Particle size, including hydrodynamic size, also plays a key role in this process, affecting the sedimentation rate, surface reactivity, and interactions with the environment [189]. It is worth noting that smaller particles may exhibit higher chemical reactivity and mobility in the environment, while larger particles may have a greater tendency to agglomerate and sediment [190]. Taken together, it can be observed that the surface properties and particle size of NPs have a significant impact on their behavior and interactions in various environments, which can have important implications for biomedical applications [191].

The initial stage of evaluating pharmaceutical systems based on IONPs involves a biodistribution study. This aims to assess the general health of the animal subjects and the interaction of the NPs with living tissue structures, as well as any potential immune response. Biodistribution studies in healthy subjects can provide valuable information on the biocompatibility and elimination mechanisms of nanomaterials, as well as their potential impact on the organism [99,192,193]. The pharmacokinetics of IONPs are relevant to medical applications such as development of drug delivery systems, where the circulation time of NPs directly influences the efficiency of the treatment [100]. The behavior of IONPs in vivo directly depends on the NPs’ physico-chemical properties, which means that it is necessary for all nanosystems to undergo in vivo preclinical testing before reaching a clinical study [101]. Mainly due to its aggregation tendency, bare IONPs can rapidly undergo opsonisation in vivo and be eliminated from the blood flow [11,194]. 

PEGylation, the process of attaching PEG to IONPs, has been demonstrated to increase the circulation time of NPs in the body [195], but it can also lead to their elimination through various pathways. Cole and colleagues [196] showed that PEG-conjugated IONPs were predominantly eliminated through splenic macrophages and less through specialized hepatic cells, a phenomenon correlated with prolonged systemic circulation time and their larger hydrodynamic size. Renal excretion of PEG-conjugated nanoparticles is less likely, as their large size prevents passage through renal fenestrations [191]. However, depending on the intended application, the internalization of NPs by macrophages can be either beneficial or undesirable. For example, in the diagnosis of certain conditions associated with increased inflammation, this aspect is desirable, but in applications such as visualizing cancerous formations or vascular angiography, it is an undesired phenomenon [197].

**Figure 10:**
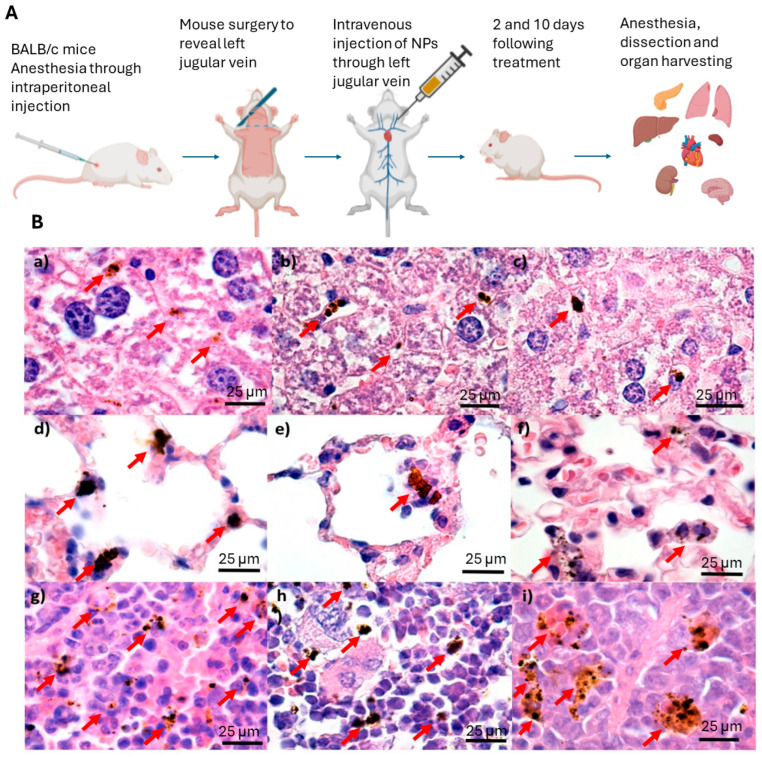
In vivo biodistribution study of PEGylated iron oxide nanoparticles (IONPs): (**A**) protocol for BALB/c mice manipulation and treatment; organ harvesting was carried out at 2 and 10 days after treatment [193]. Scheme created with Biorender.com (accessed on 15 March 2024) [49]. (**B**) Transversal sections through the liver (**a**–**c**), lung (**d**–**f**), and spleen (**g**–**i**) of mice injected with IONPs after 2 days; hematoxylin–eosin staining, magnification: 1000×, scale bar: 25 µm. Red arrows indicate the locations of nanoparticle aggregates, which appear like dark brown aggregates. Image adapted from Ref. [192].

Our group has investigated the influence of the PEG molecular weight on the biodistribution of the IONPs (Fe_3_O_4_, Fe_3_O_4_@PEG20K, Fe_3_O_4_@PEG35K) through a histological preparation of tissue samples collected from the main organs [192] (Figure 10A). IONPs were administered intravenously with a dose of 4 mg nanoparticles/kg to mouse models, and the results were compared at 2 and 10 days after administration (Figure 10A). It is worth noting that, following intravenous administration, NPs were not detected in the brain, myocardium, or pancreas. However, macrophage cells in the liver, spleen, and lungs contained IONPs, and only minimal histopathological changes were detected. The clearance of NPs primarily occurs via the single-cell phagocytic (reticuloendothelial) system, with a particular focus on the liver, spleen, and, to a lesser extent, the lungs. It should be noted that PEGylation of IONPs extends their circulation time in the blood and affects their clearance pathway [192]. Macrophages in different organs exhibit varying capacities for uptake of IONPs, which could significantly impact the development and application of this type of NP in medicine. Therefore, it is important to pay special attention to the desired clinical applications [192].

Figure 10B displays cross-sectional images of the liver (Figure 10B(a–c)), the lungs (Figure 10B(d–f)), and the spleen (Figure 10B(g–i)) of mice injected with NP with different coatings. According to the investigations, the IONPs were found to be localized in Kupffer cells, reticular macrophages in the liver, and macrophages in lung alveolae, which suggests that opsonization facilitates their removal from the blood [191] (Figure 10). Moreover, according to recent research, it has been suggested that macrophages in the spleen may play a crucial role in filtering IONPs, thereby acting as a secondary filtration barrier. Furthermore, the presence of IONPs in the lungs can be attributed to the excess of NPs in the bloodstream. According to the study, it has been observed that IONPs tend to accumulate in the red pulp of the spleen (Figure 10B(c)). This could be attributed to the transportation of the NPs by macrophages through the porous capillaries in those regions. Macrophages in the pulmonary, hepatic, and splenic regions have been found to have varying abilities to capture NPs, which may be attributed to differences in the intensity of their reactions to specific antigens present in the local environment.

## 5. Conclusions

To further our understanding of the role of IONPs in nanomedicine, it is important to investigate their internalization process. This will assist in the refinement of more accurate and focused delivery methods. NPs are taken up by cells through a variety of mechanisms, such as phagocytosis, pinocytosis, and caveolae- or clathrin-mediated endocytosis. These mechanisms are commonly utilized in nanomedicine to transport drugs and other bioactive molecules to target organelles in cells. Recent advancements in material characterization technologies have significantly enhanced our comprehension of the intricacy of this phenomenon and our capacity to regulate cellular interactions, which is crucial for reducing potential adverse effects on healthy tissues.

Our group has conducted extensive research in this area, contributing to the development of drug delivery systems for radiosensitization of tumor cells using IONPs (IONP_CO/DOX_). In addition, we have studied the internalization mechanism of these nanoparticles into 2D and 3D biological structures using various techniques, such as microscopy and spectroscopy.

According to the analysis of the literature, it can be inferred that the properties of IONPs have an impact on the mechanism of internalization. For instance, a size of approximately 50 nm is found to promote internalization by effectively activating membrane receptors. Moreover, certain cell types can benefit from the absorption enhancement provided by specific shapes, such as nano-brittles. The positive charge of the NP surface also stimulates internalization in a dose-dependent manner. Additionally, NPs with hydrophobic surfaces are more easily absorbed by cells. Lastly, the rigidity and functionalization with specific groups can affect the internalization mode and cellular behavior. The conclusions drawn from this research emphasize the importance of carefully aligning the characteristics of NPs with their biological targets due to the complexity of the process.

The investigation of the internalisation process of IONPs in two- and three-dimensional cell cultures required a detailed analysis of the methods used. Several techniques for NP characterization are discussed and presented, along with methods to visualize NP–cell interactions using microscopy and spectroscopy.

The microscopy techniques suggest that the cells absorbed IONPs through endocytosis without affecting cell morphology. Fluorescence microscopy was helpful in identifying the NP localization in relation to the tumor cells’ nuclei. Dark-field microscopy provided a rapid and non-invasive method of detecting NPs in biological structures. Electron microscopy is a valuable technique for providing high resolution information regarding the interaction of NPs with cell organelles. Moreover, scanning electron microscopy provides information on the nanobiointerface created between the IONPs and the tumor cells. 

In addition, spectroscopic techniques, such as inductively coupled plasma mass spectrometry (ICP-MS), energy-dispersive X-ray spectroscopy (EDX), and particle-induced X-ray emission (PIXE) spectroscopy, are also crucial for characterizing NPs and evaluating their interactions with cells. The article presents an in-depth examination of the distribution and quantity of NPs within cellular structures. 

In the case of three-dimensional in vitro cultures, they are used in combination with immunohistochemical techniques and represent a powerful approach for investigating cellular interactions with NPs and evaluating their therapeutic efficacy in the treatment of cancer and other conditions, using in vitro preclinical models able to better mimic conditions in the human body such as architecture, cell composition, and phenotype. However, it is widely acknowledged that preclinical in vivo studies are essential to validate the efficacy and safety of NP therapy, as changes in particle size and surface area can have a significant impact on their behavior in the body.

In conclusion, based on literature studies and our own results, it appears that the internalization of IONPs, such as IONP_CO/DOX_, is a complex yet effective process for creating safer and more precise drug delivery systems. This has the potential to reduce harmful side effects on normal cells.

## Figures and Tables

**Table 1:** The influence of the properties of nanoparticles depends on the internalization method.

NPs Property	Parameter	System Description	Cell Type	Internalization Mechanism	Method	Observation on Internalization Efficiency	Reference
Size	10 nm 70 nm 200 nm	protoporphyrin IX/IONPs	RAW264.7 cells	Endocytosis	FM	70 nm NPs > 10 nm or 200 nm NPs ← more active in stimulating membrane receptors.	[62]
	60 nm, 110 nm, 142 nm	IONPs @ APTES, DMSA, AD	HeLa cells	Energy-dependent endocytosis	OM	Lower-hydrodynamic-diameter NPs > high-hydrodynamic-diameter NPs ← require less energy.	[63]
Shape	Spheres, Bricks	IONPs	bEnd.3 cells	Caveolin-mediated endocytosis	[64]	Bricks > spheres ← interference with the caveolae.	
	Spheres, Cubes, Plateles	IONPs	FaDu cells	Endocytosis	[65]	High-length IONP cubes > spheres and platelets ← they form aligned clusters.	
Surface charge	Cationic, anionic	IONPs—CHIT, DEX, PAA, PEG, PC	A549 cells	Endocytosis	CM and TEM	Cationic IONPs > anionic IONPs.	[66]
	Cationic, anionic	IONPs @ APTES, DMSA, AD	HeLa cells	Energy-dependent endocytosis	OM	Cationic IONPs > anionic IONPs in HeLa cells.	[63]
	Cationic, anionic	IONPs @ aminoPVA, OA	HT-29 and Caco-2 cells	Not studied	OM	Cationic IONPs > anionic IONPs in 2D cell models. Cationic NPs invade HT-29, Caco-2 3D cell spheroids. Anionic NPs invade only Caco-2 spheroids. None of the NPs cross the 3D membrane models.	[67]
	Cationic, anionic, neutral	Not described	RAW264.7 cells	Not studied	UV-VIS	Cationic and anionic IONPs > neutral IONPs ← non-specific electrostatic interactions with membrane proteins.	[68]
Hydro-phobicity/Hydrophilicity	Hydro-philic	Hydrophobic Core- hydrophilic shell NPs of PLGA, PLGA@ CHI, PLGA@ PF68, PLGA@ GEL, PLA@ GEL, PCL@ GEL loaded with coumarin	In vivo biodistribution in mouse eye model	Passive transport	FM	Hydrophilic NPs → follow the conjunctival pathway in the eye → pass from clear to the iris–ciliary body through vessel uptake.	[69]
	Hydro-phobic	IONPs@ MPS	HAoECs	Endocytosis	OM and FM	MPS-coated IONPs are internalized in HAoECs ← absorption through the plasma membrane is facilitated by the hydrophobic NPs.	[70]
Rigidity	Stiffness	GM3- lipid- PLGA-PLA NPs	CD169, expressing macro-phage cells	Actin- dependent phagocytosis	FM	NPs with the stiffest cores are internalized in a higher manner in activated macrophages.	[71]
	Stiffness	ALG@ lipidic bilayer	MDA-MB-231, MCF7, MCF10A cells	Not studied	FM	NPs with the highest stiffness are internalized in a lower manner in breast cancer cells.	[72]
Functional groups	OH, NH_2_, COOH	IONPs @ BSA, PEG	A549 cells	Clathrin- mediated endocytosis and caveolin- mediated endocytosis	FM	BSA-coated IONPs are internalized via clathrin-mediated endocytosis ← (NH_2_) and (COOH). PEG-coated nanoparticles are taken up via caveolin-mediated endocytosis ← (OH).	[57]
	OH	IONPs @ SiO_2_, DEX	HMDM, MDDC cells	Active actin cytoskeleton- dependent mechanism	TEM	IONPs@ SiO_2_ > DEX- coated IONPs ← the coating material can affect the protein interaction.	[73]
	OH	IONPs/PLGA/Cy5.5	MSCs cells	Clathrin- mediated endocytosis	FM	SPION-clustered PLGA with average hydrodynamic size of 115.2 nm and negative charge are internalized in MSCs.	[74]
	COOH, NH_2_	IONPs@ amphiphilic polymer terminated with (COOH) or (NH_2_) groups	HCAEC cells	Vesicle- mediated	TEM, ICP-MS	Both types of nanoparticles are internalized through vesicles. IONPs with (COOH) > IONPs with (NH_2_) groups.	[75]

Abbreviations: fluorescence microscopy (FM), optical microscopy (OM), transmission electron microscopy (TEM), confocal microscopy (CM), iron oxide nanoparticles (IONPs), aminopropyl-triethoxy silane (APTES), Dimercaptosuccinic acid (DMSA), Aminodextran (AD), chitosan (CHI), dextran (DEX), polyacrylamide (PAA), polyethylene glycol (PEG), phosphatidylcholine (PC), polyvinylamine (aminoPVA), oleic acid (OA), polylactic-co-glycolic acid (PLGA), Pluronic F68 (PF68), polylactic acid (PLA), gelatine (GEL), polycaprolactone (PCL), 3-methacryloxypropyl trimethoxysilane (MPS), alginate (ALG), bovine serum albumin (BSA). “>” states for “internalized in higher amount”, “<” states for “internalized in lower amount”, “→” states for “determines”, and “←” states for “is determined by”.

## Data Availability

No new data were created or analyzed in this study. Data sharing is not applicable to this article.
